# Turnover in close friendships

**DOI:** 10.1038/s41598-022-15070-4

**Published:** 2022-06-30

**Authors:** Chandreyee Roy, Kunal Bhattacharya, Robin I. M. Dunbar, Kimmo Kaski

**Affiliations:** 1 Department of Computer Science, Aalto University School of Science, Espoo, Finland; 2 Department of Industrial Engineering and Management, Aalto University School of Science, Espoo, Finland; 3 Department of Experimental Psychology, University of Oxford, Oxford, UK; 4 The Alan Turing Institute, London, UK

**Keywords:** Behavioural methods, Computational science, Statistics

## Abstract

Humans are social animals and the interpersonal bonds formed between them are crucial for their development and well being in a society. These relationships are usually structured into several layers (Dunbar’s layers of friendship) depending on their significance in an individual’s life with closest friends and family being the most important ones taking major part of their time and communication effort. However, we have little idea how the initiation and termination of these relationships occurs across the lifespan. Mobile phones, in particular, have been used extensively to shed light on the different types of social interactions between individuals and to explore this, we analyse a national cellphone database to determine how and when changes in close relationships occur in the two genders. In general, membership of this inner circle of intimate relationships is extremely stable, at least over a three-year period. However, around 1–4% of alters change every year, with the rate of change being higher among 17-21 year olds than older adults. Young adult females terminate more of their opposite-gender relationships, while older males are more persistent in trying to maintain relationships in decline. These results emphasise the variability in relationship dynamics across age and gender, and remind us that individual differences play an important role in the structure of social networks. Overall, our study provides a holistic understanding of the dynamic nature of close relationships during different stages of human life.

## Introduction

Human societies are built up in part out of personal social networks. These networks, consisting of nodes as individuals and edges representing relationships between them, evolve through the addition and deletion of nodes, reflecting the formation of new bonds and the dissolution of old ones. We know relatively little about the rates of churn in networks and the dynamics of relationship change – despite the fact that these are fundamental aspects of social networks. Previous research has shed some light on the temporal evolution of relationships that are manifestations of different stages of human life ^1–3^, geography^4^, structural changes in personal networks^5^ and patterns of mixing^6^, among others^7–10^. In this paper we examine the dynamics of relationship growth and decay with respect to ego age and gender using real world data from mobile phone records. We compare these turnovers with respect to different types of relationships. Analysing communication patterns through such mobile phone datasets is a valuable way to gain insight into the social networks of humans^3,11,12^ along with their behavioural patterns caused by seasonal and geographical changes^4,13^, daily rhythms^14–16^ and mobility patterns^17,18^ among others ^7,19–21^.

Human social networks have a layered structure, defined by the fractal structure of the Dunbar number^22^. This fractal structure forms a series of layers in the network that reflect the emotional closeness of the individuals involved^23–26^. Studies on networks formed through social media, such as Facebook and Twitter have shown layered structures similar to offline face-to-face networks ^27^, and similar patterns have been reported from massive multiplayer online games ^28^. The numbers 1.5, 5, 15, 50, and 150^29^ cumulatively represent the number of intimate friendships, close friendships, best friends, good friends and generic friends, respectively. Note that we use the term ‘friends’ here in a generic sense to include extended family as well as more conventional friends. Friends may be lost or gained in each of these layers^30,31^. The size of egocentric networks has been observed to be higher for younger people and becomes more stable as they grow older^2,32^. The emotional closeness of an ego or focal individual with its alters (the individuals connected to the ego in each of these layers) is inversely proportional to the number of alters in the layer, with the innermost layer of around 5 alters (usually known as the support clique) consisting of the most intimate friendships and are the closest of all^24,33,34^. This layer commonly includes parents, siblings or close friends as well as romantic partners from the first layer and is the set of people the ego would rely on for advice, help and emotional support. The ego invests most of her or his social time in this group of alters (or close friends), with the ~ 5 members of this layer accounting for ~ 40% of ego’s total social time^31^.

**Figure 1:**
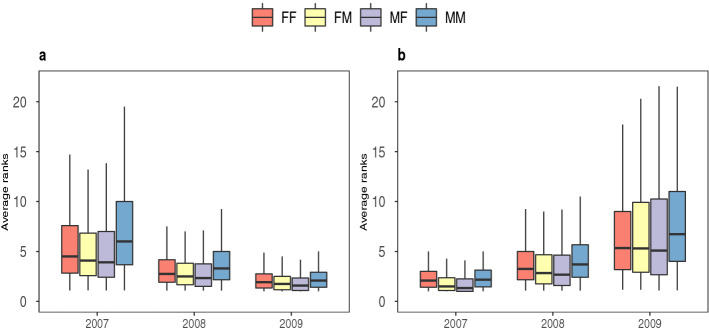
Change in the rank of alters during the formation or decay of ego-alter relationships over a 3 year period. The ranks have been calculated monthly, then averaged over 12 months for each year and a summary of their progressive increase/decrease over a span of three years are plotted in a boxplot for (**a**) the set of ego-alters forming close relationships and (**b**) another set of ego-alters decaying from close relationships. The black horizontal lines in the middle represent the median of the distribution. The box plot includes all the values within the range of the 25th and 75th percentile and the ends of the whiskers represent the maximum and the minimum ranks excluding outliers. The red box represents ranks of females and blue box represents the same for males in their respective same-gender egocentric networks. The yellow box represents the ranks of males in female egocentric networks and violet box represents the same for females in a male egocentric networks. We find that for all years in both cases, a male’s rank among his male friends is consistently lower than other groups indicating that female friendships and opposite-gender friendships generally form stronger bonds than male friendships.

In our study, we examine the closest relationships (“the support clique”) of the friendship network by studying their calling activities through mobile phone communication data of a particular service provider from a southern European country^7^. This unique mobile phone dataset contains call detail records (CDRs) and demographic information of anonymized users that have remained loyal to the service provider for a period of three years^19,35^. Survey data based on questionnaires to study social networks provide a more realistic approach but are limited by the subjectivity of answers, bias in recollection and the number of individuals included for the studies. Mobile phone datasets have become a popular way of studying large-scale social networks^36^ and can provide a more objective view on communication patterns^35,37^. However they are inevitably limited by the fact that they ignore other modes of communication (face-to-face interactions, social media and landline communications among others)^38^. The dataset used in the current study predates the popularity of smartphones as well as most social media and since mobile phone numbers are usually exchanged between trusted members of a community, we can reasonably assume that communications include a sub-network of known individuals. More importantly, perhaps, it has been demonstrated that online ties correlate with offline ties since friends tend to communicate with friends in proportion to their emotional closeness irrespective of the medium of communication^27,38^. While both approaches to studying social networks have their pros and cons, the ability to study large scale data helps us to gain insight into the network at a macroscopic level. Phone call datasets provide us with very large samples, thus enabling us to study significant events such as relationship formation and decay that normally occur at low frequencies and are hence difficult to study using more conventional self-report methods. It is important to be clear that our concern focuses on understanding the nature of the relationship itself, not the mode of communication whereby the relationship is maintained. A mobile phone calling dataset provides us with a convenient and rigorous way of assessing relationship quality simply because it requires two individuals to be willing to invest time in each other.

Many studies of social networks focus either on the overall top-down properties of networks (e.g. centrality, bridging) or on the triadic structure of relationships. Our focus, however, is on the properties of individual ties, and for this a different bottom-up approach that focuses on the individual ties themselves rather than on the properties of sets of ties. Note also that we are not concerned here to evaluate the causes of relationship change. We are simply concerned to determine how frequently close relationships change, and whether this differs between the two genders and across major age cohorts among adults. Also we note that here, we are not concerned about changes in the relationships of under-17s due to not having enough data in this age cohort.

In the present study, we aim to determine two things: first, how frequently do relationships within this core grouping change and, second, how does this vary with age and gender. Since phone calls involve a commitment of time, a high rate of calling activity between a pair of individuals can be reasonably assumed to indicate a close relationship between them, while a lower calling rate would indicate a weak tie. By the same token, we interpret a decline in calling rate as indicating the termination of the relationship. If the decline is sudden (a step like change in call frequency), we interpret this as indicating a falling out. If call frequency declines more gradually, it suggests a gradual loss of interest.

## Results

### Ranks of subscribers

In this study we have considered only those relationships between pairs of mobile phone users that form a close bond over a span of three years or which decay from a close bond (see Methods). A pair of service subscribers is considered to have a close bond between them if both of them are in the top five rank of each others’ social networks. By definition, these two users fall within the first two Dunbar layers of their respective networks. The ranks of users in all pairs have been calculated in their respective egocentric social networks and are grouped according to their gender (see the Methods section). The same-gender interactions are termed as female-female (FF) and male-male (MM) ego-alter friendships while the opposite-gender interactions are termed female-male (FM) and male-female (MF) ego-alter friendships, respectively.

Figure 1 gives a summary of the ranks, in the form of a box plot, for males and females in their respective friend or partner’s networks from the year 2007 to 2009. Formation of a close bond is represented by increased calling activity between the pair and a shift from a lower to a higher rank over time (within the range ranks 1–5, where rank 1 is assigned to the individual called most often). The set of ego-alter pairs that show a progressive increase in ranks in successive years are shown in of Fig. 1a. The colour red/blue represents ranks of females/males in their respective female/male partner’s egocentric network. For opposite gender friendships, the colours yellow/violet represent ranks of males/females in the female/male partner’s egocentric network. The ranks of the males in same gender friendships are observed to be noticeably lower than all the other groups for all three years indicating that males might not form as close bonds with their male counterparts when compared to female-female or even opposite-gender friendships.

We observe the same trend in Fig. 1b where we exhibit the ranks for only those ego-alter relationships that have decayed from a close bond by a gradual decrease in calling activity and a decrease from higher to lower ranks over a span of three years. These decaying relationships also include pairs whose ranks decrease to lower values but still remain ≤ 5. The extent of a fall or rise in ranks in the years 2007 → 2008 and 2008 → 2009 is also greater in the case of male-male friendships, indicating that male friendships are not as close as opposite-gender or female-female friendships (see Supplementary Fig. 1).

### Network turnover

**Figure 2:**
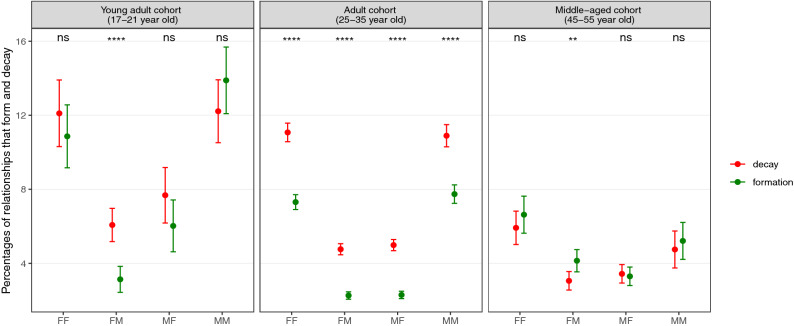
Percentages of ego-alter relationships that have formed or decayed from a close bond among peers in three different age cohorts. Total number of pairs in each group are given in Supplementary Table I. The error bars are shown with 95% confidence level and the p values from two proportions z-test are shown in the top of each panel to illustrate how significantly different the formation and decay percentages are. We have used the following notation scheme to represent the significance levels of p values: not significant (ns) for *p* > 0.05, and significant to the degree of (*) for *p* ≤ 0.05, (**) for *p* ≤ 0.01, (***) for *p* ≤ 0.001 and (****) for *p* ≤﻿ 0.0001. Overall the percentage-wise formation and decay of friendship for the young-adult cohort are observed to be higher than those for the adult and middle-aged cohorts. We see a marked difference in the behaviour of female ego’s relationships with their male alters with low values of formation and higher values of decay in the young-adult and adult groups. Moreover, we observe that young adult male egos have the comparatively same values of formation and decay with female alters but change their behaviour in the adult group. The percentage values corresponding to the graph are shown in Supplementary Table III.

**Figure 3:**
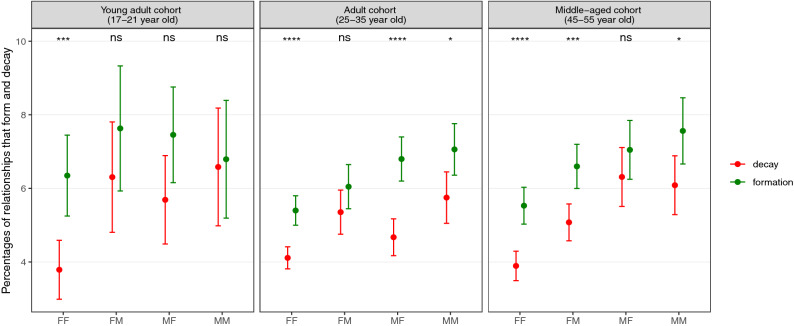
Percentages of ego-alter relationships that have formed or decayed from a close bond among non-peers in three different age cohorts. Total number of pairs from each group are given in a corresponding Supplementary Table II. The error bars are displayed with 95% confidence level and the p values from two proportions z-test are shown in the top of each panel to illustrate how significantly different the formation and the decay percentages are using the same scheme as mentioned in Fig. 2. Here, we find that the percentages of formation are comparatively higher than percentages of decay for adult and middle-aged cohorts. Particularly, the FF group has a higher percentage of formation than decay in all age groups. The corresponding percentage values for this graph is shown in Supplementary Table IV.

In order to investigate the turnover in friendship networks we compared the calling activities between three different age groups: young adults (from 17 years to 21 years), adults (from 25 years to 35 years) and the middle-aged (from 45 to 55 years). The young adult cohort consists of individuals that go through major changes in their lives due to education or starting work, whereas the adult cohort spans the period associated with marriage and reproduction (note that divorce and re-marriage is less common in this Catholic country), and the middle-aged cohort identifies that period in life when the social focus is mainly centred around older children, grandchildren and the extended family. When the age difference between an ego and an alter is less than or equal to 10 years, then we deem it a “peer” interaction and if the difference is between 20 to 40 years of age then we deem it to be a “non-peer” interaction. The total number of pairs in each of the gender and age based groups are given in Supplementary Tables I and II for peer and non-peer interactions, respectively. We then selected only those pairs that either form a close bond or decay from a close bond according to their calling activity as described in the methods section. Figure 2 summarises the percentages for peer interactions and Fig. 3 those for non-peer interactions.

Overall, the low frequencies with which close relationships form and decay in a three year period is an indicator of how stable these inner core relationships typically are. Nonetheless, there are some significant patterns in these data. The percentage of relationship formation (3.1 ± 0.7%) in opposite gender friendships for the female egos belonging to the young adult cohorts is almost half of their corresponding decay percentage (6.1 ± 0.9%): in effect, they lose close opposite gender relationships over time without replacing them, although the rate at which this happens is low (roughly 1% of relationships per year). In contrast, the male egos in the young adult cohort have similar values for both formation (6.0 ± 1.4%) and decay (7.7 ± 1.5%) indicating that they make friends as easily as they lose them. A similar pattern is observed for female egos in the adult cohort as well for opposite gender relationships. The rates at which relationships form (2.3 ± 0.2% for both male egos and female egos) in the adult cohort are almost half of their decay percentages (4.8 ± 0.3% and 5.0 ± 0.3% for female and male egos, respectively). Thus, the male and female egos behave similarly in the adult group but behave differently in the young adult group.

For same gender friendships, we observe that the formation and decay percentages balance each other out in the young adult group for both female (10.9 ± 1.7% and 12.1 ± 1.8% for the formation and decay, respectively) and male (13.9 ± 1.8% and 12.2 ± 1.7% for the formation and decay, respectively) egos, with rates of turnover that are higher than in the adult and middle-aged cohorts. We also observe that they have a distinctively higher decay than formation rates, particularly in the adult group with the percentages of the formation being  {7.3 ± 0.4%, 7.7 ± 0.5%} and the percentage of the decay being {11.1 ± 0.5%, 10.9 ± 0.6%} for female and male egos, respectively. For the middle-age cohorts the percentages of the relationship formation and decay for all gender groups are in balance with values of the formation being {6.6 ± 1.0%, 5.2 ± 1.0%, 4.1 ± 0.6%, 3.3 ± 0.5%} and the decay being  {5.9 ± 0.9%, 4.7 ± 1.0%, 3.1 ± 0.5%, 3.4 ± 0.5%} for FF, MM, FM, MF friendships, respectively, where the gender on the left is the ego and gender on the right is the alter. In other words, the turnover is high in the younger cohorts in all gender groups and becomes smaller as individuals age.

When the users interact with their non-peers (see Fig. 3), we observe that rates of formation are higher than decay for all gender groups in the adult and middle-aged cohorts indicating that they try to maintain their non-peer relationships. Particularly, we find that the percentage of relationships formed is significantly higher than the number that decay in FF group for all age cohorts. The percentages of formation are {6.3 ± 1.1%, 5.4 ± 0.4%, 5.5 ± 0.5%} and percentages of decay are {3.8 ± 0.8%, 4.1 ± 0.3%, 3.9 ± 0.4%} for young adult, adult and middle-aged cohorts, respectively, suggesting that female egos form increasingly closer bonds with their older female alters in all age groups.

### Temporal pattern of calling activity

**Figure 4:**
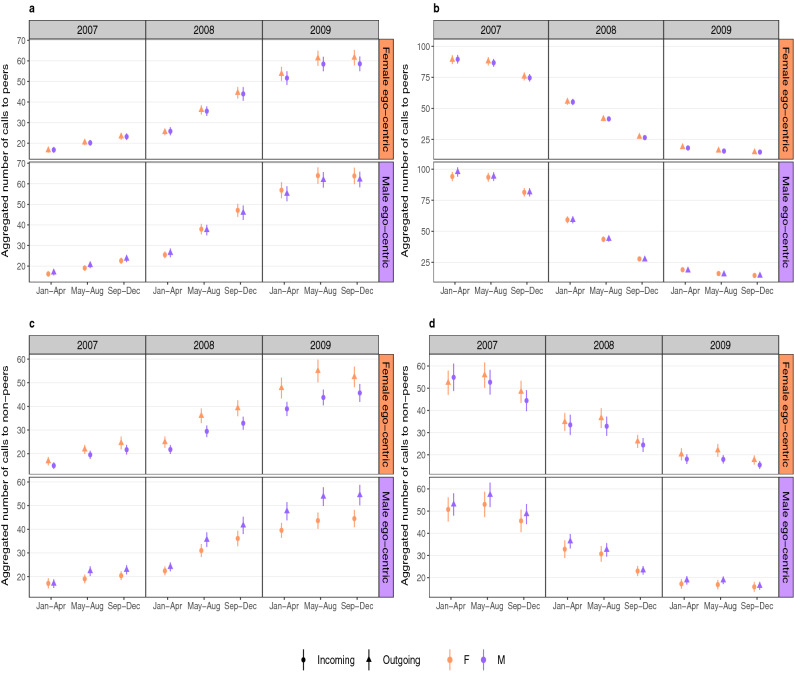
Calling activities of adult (25–35 year old) cohorts between peers and non-peers and having interactions with their opposite genders. The aggregated calls over four-month intervals over a span of three years have been calculated for the opposite-gender friendships for peers (having age differences of less than 10 years) exhibiting (**a**) formation of close bonds and (**b**) decaying of close bonds. We also show the aggregated calls of the young age cohorts calling their non-peers (having age differences of 20 to 40 years) for formation (**c**) and decay (**d**) of close bonds. The orange/violet coloured panels on the right of each of the graphs represent the calls made in a female/male egocentric network. In all the graphs, filled circles/triangles represent incoming/outgoing calls and orange/violet coloured shapes indicate calls initiated by female/male egos in the egocentric networks. Therefore, an orange coloured triangle indicates calls made by females to males while violet coloured circles represent calls made by males to females in a female egocentric network. Similarly, orange coloured circles and violet coloured triangles indicate female-initiated and male-initiated calls in a male-egocentric network. The female-initiated calls in both male and female egocentric networks in the year 2009 show systematically higher values than male-initiated calls in the case of formation of close relationships between peers while there is not much difference visibly between them in decaying relationships. We also find that younger cohorts are more active than their older cohorts in terms of calling activities since they initiate distinctly more number of calls than their older counterparts when forming a close bond. For decaying relationships, we do not find much difference in the calling activities of both genders in female egocentric networks but in the male egocentric network we find that male initiated calls are slightly more female initiated ones.

To explore these patterns in more detail in the adult cohort, Fig. 4a and b exhibit the calls made to peers aggregated in four month blocks for the formation and decay of close bonds, respectively. Figure 4c and d represent the formation and decay of close bonds through calls made to non-peers. The decaying relationships for adult cohort calling peers (see Fig. 4b) do not show much difference between calls initiated by males and those initiated by females. However, when they form a close bond, even though calls initiated by both genders are not significantly different from each other (i.e. p values > 0.05 obtained from paired t-tests), there is a striking tendency for calls made by females to their male counterparts to increase faster than those made by males, suggesting that females are more proactive than males in building opposite-gender relationships (see Fig. 4a). We also observe in Fig. 4c that both genders call their older counterparts more often when they are trying to form a close bond with their elders (to a significant degree as p values < 0.05 obtained from paired t-tests), which suggests that younger individuals are more active in initiating the relationship. In a decaying relationship, we notice that there is not much difference in the number of calls initiated by both genders in a female egocentric network, but in the male egocentric network the younger males systematically call older females more than they receive calls from them. The corresponding graphs for the young adult cohort is displayed in the Supplementary Fig. 2. We observe similar calling patterns for all interactions except in the case of decaying relationships in female egocentric networks where male-initiated calls were higher than female-initiated calls.

**Figure 5:**
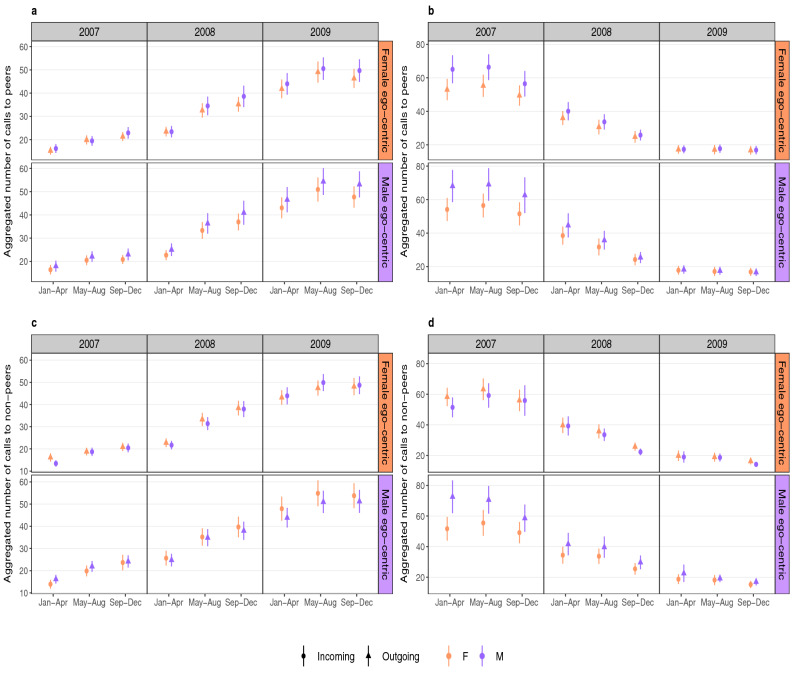
Calling activities of middle-aged age (45–55 year old) cohorts between peers and non-peers and having interactions with their opposite genders. The aggregated calling activities of middle-aged cohorts between their peers (having age differences of less than 10 years) has been shown in (**a**) and (**b**) along with non-peers (having age differences of 20 to 40 years) in (**c**) and (**d**). The plots on the left exhibit those relationships that form a close bond and the plots on the right exhibit decay of close bonds. The orange/violet coloured panels on right of each of the plots represent female/male egocentric network. The filled triangles/circles represent outgoing/incoming calls and orange/violet colour of the points represent calls initiated by females/males in their respective networks. We find that middle-aged individuals calling their peers have slightly higher male-initiated calls than females in both forming and decaying relationships. Their calling activity with non-peers show a consistent behaviour with the adult cohorts’ interaction with non-peers in Fig. 4c where they are receiving slightly higher number of calls from the younger opposite gender individuals. However, we find that when relationship decays the males are making higher number of calls to the younger females.

Figure 5 shows the calling activities of the middle-aged cohorts to their peers (Fig. 5a and b) and their non-peers (Fig. 5c and d). In the case of middle-aged cohorts calling their peers, we observe that in both formation and decay of close bonds (Fig. 5a and b, respectively) males initiated more calls than females, indicating that males take the more active part in trying to maintain a relationship. In the case of the middle-aged cohorts calling their non-peers during the formation of close bonds (Fig. 5c), we observe that they are being called more by their younger counterparts of opposite gender as was the case in Fig. 4c. For decaying relationships in Fig. 5d, we again observe that the older males call more than receive calls from their younger female counterparts. We also investigated gradual and abrupt fall of calling activity in decaying relationships by looking at the differences between their monthly calling activities. However, there were no significant differences in the patterns of behaviour between these two types of relationship decline.

## Discussion

In this study we have analysed population level mobile phone communication data from a Southern European country to investigate relationship changes in different age cohorts. Firstly, in same gender relationships we observe that females tend to have higher ranks in female egocentric networks compared to males in male egocentric networks. This observation is consistent with known qualitative differences in same gender communication^20,39,40^ In general, the females tend to have fewer and more close relationships than males as can be seen through the comparison of the ranks in Fig. 1. A number of other gender differences may be noted. The median of the ranks in opposite-gender friendships has a systematic trend of females having higher ranks in their male counterpart’s network than the male’s rank in their male counterpart’s network. This suggests that men tend to give more importance to the women (spouse, mother or romantic partner) in their lives than to their male friends. This has not previously been noted in sociological studies of close relationships. We also notice that the male’s rank in their female partner’s network is comparable to the rank of females in the same gender (FF) friendships. This is consistent with the finding that females tend to have two special friends in their innermost core (a best friend who is usually a female and a romantic partner) while men tend to have only one special friend (either a romantic partner or a male friend, but rarely both at the same time)^29^.

Secondly, the overall low percentages for the formation/decay of relationships in the network as observed in Figs. 2 and 3 for mutual top 5 ranked alters that form the support clique of the egocentric network reflect a reassuring fact that close friendships typically remain stable and rarely involve fall-outs between them. It is known that this core layer of the network comprises parents, romantic partners and best friends and therefore, these important relationships might be expected to be relatively resilient to the kinds of circumstances that commonly destabilise relationships (e.g. loss of trust).

Thirdly, the percentages of the formation/decay of close relationships are higher in the young adult and adult cohorts than the middle-aged cohort, indicating more stable friendships in the latter. The young adults in this age group are usually more mobile, socially and economically, and this may be expected to impact on the stability of their relationships. We notice that, particularly for opposite gender friendships, the females have a lower rates of formation than decay, indicating that they may be more meticulous in choosing male friends^11,41–43^. The same pattern is not observed in the case of young adult male egos. This could also be due to the fact that guardianship of the family over young adult females probably tends to be exercised over a longer period than young adult males in a society^44^.

Fourthly, marked differences in the percentages of relationship formation and decay in the case of 25-35 year old adults for opposite gender friendships for both the male and female egos likely reflects the impact of reproduction^45^: in this population, the mean age at marriage at the time the phone data were sampled was 29 years, with first reproduction following soon afterwards^46^. Moving out of the parental home to establish an independent home life, combined with the arrival of children, marks a major social transition and upheaval^47,48^. We also observe that the percentages of the formation and decay eventually balance out in the middle-aged cohorts thus indicating that this age group has achieved some degree of stability in their social lives.

Finally, we observe from Fig. 3 that most relationships in all gender and age groups with non-peer interactions have higher percentages of relationships that form than decay, particularly so for the FF group in which we see consistently higher values for formation than decay. This could reflect the fact that the young adults are in the process of moving out of their parental home resulting in increased calling activity with their parents. The adult cohort also has higher percentages of formation than decay. This could be the result of their hitting the reproductive stage of life, which leads them to retain only those non-peer relationships (like aunties and others) that help them in tasks like child-rearing. Another reason for the higher rates of relationship formation in the middle-aged group could also be due to the grandmothering effect^40^ that comes into play when the mothers start taking an especial interest in their children’s lives when grandchildren come along^49^.

Additionally, we observed differences in the calling activity patterns of the opposite-gender peer and non-peer interactions among the adult and middle-age cohorts during the formation and decay of relationships. Our analyses show that after a close bond is formed between opposite gender peers in the adult cohort, it is the females that systematically tend to call their male counterparts more often. However, when the middle-age cohorts interact with their peers, then the males call their female counterparts more for both the cases when relationships are being formed and when they are decaying. Since we do not expect these kinds of relationships to happen between siblings, this could probably represent romantic or platonic friendships. In addition, we observe a distinctly higher call rate by the adult cohorts to their older counterparts when relationships are being formed. The formation of relationships in this group may be indicative of those being formed between an individual and their in-laws might reflect the fact that the adult cohort makes more effort in trying to establish a relationship with their older counterparts since younger age groups are generally more active technologically than older age groups.

Since, all the users are from the same service provider that had a 20% market share in the country, this dataset may only capture a proportion of the true support clique. However, irrespective of whether this is the case, our focus is on the set of closest relationships that an ego has rather than a specific number. Precisely how we define this subset is less important than that our sample represents a small inner core of an individual’s total network. That said, any tendency for friends and family to gravitate towards the same service provider as a result of special deals or local availability, etc., will increase the likelihood that we capture their actual network 5-layer. Indeed, analyses of the structure of complete egocentric networks from this dataset without making any presumptions about structure yield a layer of exactly this size^25^. While longitudinal studies using questionnaire-based data through Add Health^50^ can portray the different types of relationships actually involved, we believe that our cohort-based study using mobile phone data provides a complementary approach that gives us insights objectively about the gender preferences and their churn on a scale that allows rare but significant events to be examined.

In conclusion, our analyses of mobile phone data for a large population establish that the inner core Dunbar layer of friendships are stable with very few relationships moving out or entering this core. In our analyses, we find that during the younger stages of adult life people mostly lose friends, with young adults having slightly more unstable friendships as compared to older adults. However, friendships seem to become more stable during middle-age after people settle down with families and stable careers. Additionally, young females tend to be picky while choosing male friends, but after they form a close bond, they tend to be slightly more active in maintaining those relationships. Even though modes of communication through social media platforms have become more prevalent nowadays, we expect churn to occur in the latter networks irrespective of the technology used to service them. This is expected as the layered structures in friendship networks are based on emotions rather than memory^22,27^.

## Methods

### Study data

The dataset used in this study was obtained from a southern European service provider consisting of mobile phone call detail records (CDRs) of the service users for a period of three years from January, 2007 to December, 2009. The call details of the users were anonymised by the service provider by attaching a unique identifier for each of the users such that the privacy of the customer remains protected and cannot be traced back to the individuals themselves. The CDRs include a list of the caller and callee with time stamps and dates. The call durations for each call are provided only for the year 2007. The users’ age and gender along with the postal code are also listed in the dataset. A total of 644,170 remained loyal to the service provider for all three years with a total of 1,143,718 unique links between them. The users that appear or disappear in the dataset within this time period of three years were not considered to avoid confusions over ties that might already have been present in an ego’s network but through a different service provider.

### Data analyses

**Figure 6:**
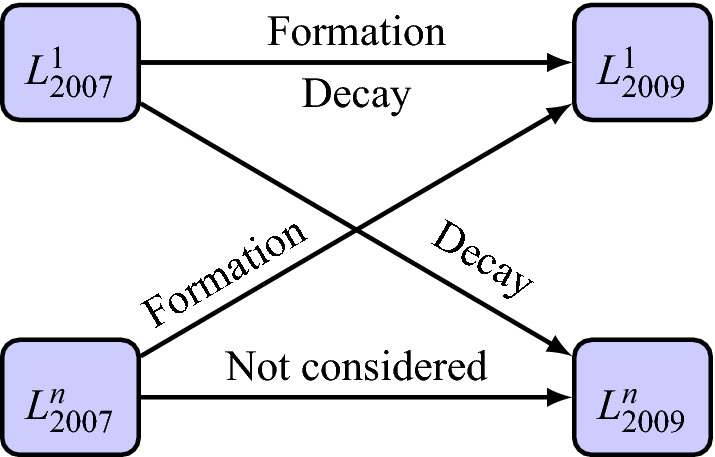
The relationships that have been considered for analyses. All pairs that were mutually in top 5 ranks in the year 2007 (represented by $L_{2007}^1$) and after a rise or decay in call activity still remain in top 5 in the year 2009 ($L_{2009}^1$) along with pairs whose ranks become lower than 5 ($L_{2007}^1 \longrightarrow L_{2009}^n$: decay where *n* in the superscript represents any rank lower than 5) or rise from a lower rank to a higher one ($L_{2007}^n \longrightarrow L_{2009}^1$: formation) have been considered. Only pairs that remain in lower ranks have not been considered. The arrows have been labelled accordingly to show the change in the ranks. Relationships having users with ranks lower than 5 in 2007 and remain low in 2009 have not been considered.

The rank of a user in another user’s network has been calculated in the following way. We consider all the alters that the user has called or received calls from on a monthly basis and have ordered them according to the total number of Incoming/Outgoing calls in a decreasing order. Alters having the highest number are ranked one and so on. The ranks are then calculated for each of the 36 months being considered and then averaged on a yearly basis. Next, we consider only those links with users having top ranks between 1 to 5 mutually in each others networks in the year 2007 and 2009. This is because we intend to study formation or decay of very close relationships that form the ”support clique” of the Dunbar layers. Thus, any pair that starts off with a mutual top 5 rank and then decays over the span of three years to lower ranks and any pair that starts of at any rank but ultimately ends with top 5 rank in the year 2009 have been considered. In such a scenario, if we represent all pairs having top 5 ranks as $L^1$ and have ranks lower than 5 as $L^n$ (where *n* in the superscript represents any rank lower than 5) then we consider only the changes in ranks of the relationships as shown by arrows in a schematic diagram in Fig. 6. The ranks could still be in top 5 even though the relationship decays or forms from a top rank to an even higher rank. The only type of relationship that has not been considered are the ones that go from $L_{2007}^n \longrightarrow L_{2009}^n$. Keeping this in mind we have a total of 178,592 links between 296,051 users with 44,115 female-female, 106,570 female-male or male-female and 27,907 male-male friendships considered for analysis and the age distribution of the users is shown in Supplementary Fig. 3. Furthermore, the interaction between users is deemed as a peer relationship if the age gap between them is less than or equal to 10 years and non-peer if it is between 20 to 40 years.

Next, we define a formation of a close relationship to occur between two pairs of users if the calling activity between them increases and yearly averaged ranks for both the users gets elevated each year:

1 $$\begin{aligned} N_{2007}< N_{2008} < N_{2009} ~\text {and}~ R_{2007}> R_{2008} > R_{2009}, \end{aligned}$$

where *N* and *R* represents the total number of calls and the numerical value of the yearly averaged ranks of the pairs of users in each others egocentric networks respectively. Similarly, a decay in relationship is defined to occur if calling activity decreases and yearly averaged rank gets demoted each year.

2 $$\begin{aligned} N_{2007}> N_{2008} > N_{2009} ~\text {and}~ R_{2007}< R_{2008} < R_{2009}. \end{aligned}$$

This filtering of the data leads to 8,637 pairs of users who end up forming a close relationship in 2009 and 10,310 pairs whose relationship decayed over three years. The age distribution of alters of the egos considered in the three different categories (young adults between 17-21 years, adults between 25-35 years and middle-aged between 45-55 years of age) are shown in Supplementary Figs. 4 and 5 for peer and non-peer interactions among the opposite gender, respectively.

## Supplementary Information

Supplementary Information.

## Data Availability

The datasets generated during and/or analysed during the current study are not publicly available due to a signed NDA but are available from the corresponding author on reasonable request.
