# Supplementary material for: Turnover in close friendships
Source: Sci Rep. 2022 Jun 30;12:11018. doi: 10.1038/s41598-022-15070-4 (PMC9247060; doi:10.1038/s41598-022-15070-4)
Supplement: Supplementary file 1 — Supplementary Information. [file 41598_2022_15070_MOESM1_ESM.pdf]

Turnover in close friendships

Chandreyee Roy,[1,*] Kunal Bhattacharya,[1,2] Robin I.M. Dunbar,[3] and Kimmo Kaski[1,4]

[1] *Department of Computer Science, Aalto University School of Science, Espoo, Finland*

[2] *Department of Industrial Engineering and Management, Aalto University School of Science, Espoo, Finland*

[3] *Department of Experimental Psychology, University of Oxford, Oxford, UK*

[4] *The Alan Turing Institute, London, UK*

I. EXTENT OF CHANGE IN THE RANKS OF THE ALTERS

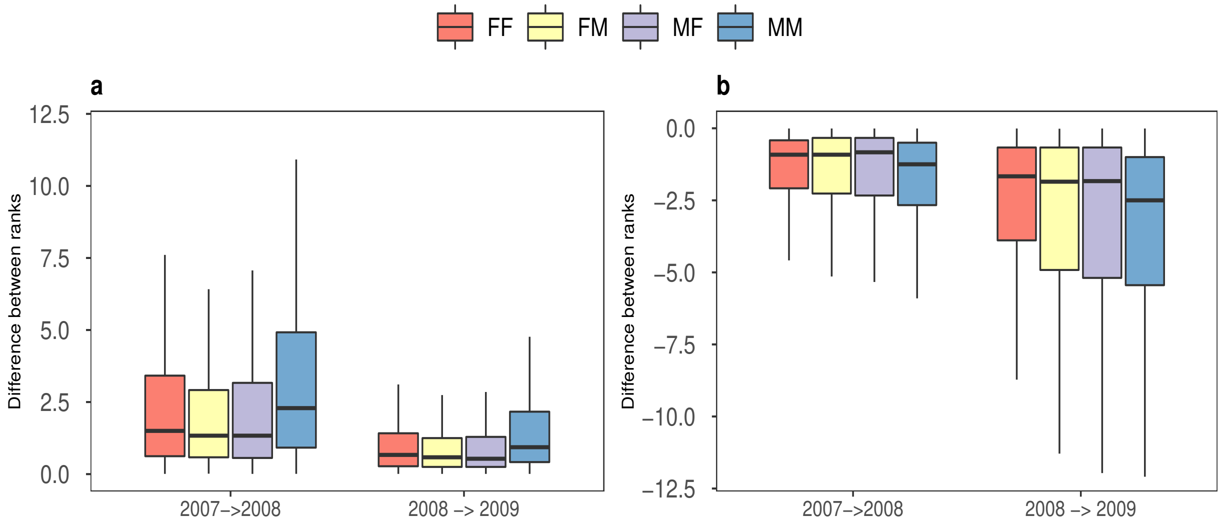

FIG. 1. **Difference in the ranks of the users for consecutive years.** The extent of change between the ranks of the alters over the years is calculated by taking the difference between the ranks in 2007 to 2008 and 2008 to 2009. (a) The difference will be positive in the case of formation of close bonds as ranks go from lower to higher and, (b) they will be negative in the case of decay of relationships as the ranks go from high to low. In both cases of formation and decay we observe that a male's rank in their male friend's network drops more than the other groups.

* chandreyee.roy@aalto.fi

II. TOTAL NUMBER OF LINKS IN EACH COHORT

The total number of links that have been considered for our analysis according to Fig. 6 in the methods section of the main text are then divided according to the age and gender groups. The following two tables contain the total number of links in each of the groups for each age cohorts.

Ego - alter relationship	Young-adult cohort (17-21 year old)	Adult cohort (25-35 year old)	Middle-aged cohort (45-55 year old)
female-female	1289	13259	2399
male-male	1498	11448	1727
female-male	2520	25290	4878
male-female	1146	25477	5216

TABLE I. **Total number of links belonging to each cohort having peer interactions.** The total number of links in each cohort that includes both relationships that form and decay along with other types of relationships as mentioned in the manuscript are displayed in this table for each of the gender based groups. The denominators for formation and decay in each ego-alter group corresponds to Fig. 2 in the main text (or Supplementary Table 3).

Ego - alter relationship	Young-adult cohort (17-21 year old)	Adult cohort (25-35 year old)	Middle-aged cohort (45-55 year old)
female-female	2032	12741	9655
male-male	957	4504	3319
female-male	983	6069	6381
male-female	1529	7383	3803

TABLE II. **Total number of links belonging to each cohort having non-peer interactions.** The total number of links in each cohort that includes both relationships that form and decay along with other types of relationships as mentioned in the manuscript are displayed in this table for each of the gender based groups. The denominators for formation and decay in each ego-alter group corresponds to the Fig. 3 in the main text (or Supplementary Table 4).

III. PERCENTAGES OF RELATIONSHIPS THAT FORM AND DECAY IN TABLE FORM

Ego - alter relationship	Young adult cohort (17-21 year old)		Adult cohort (25-35 year old)		Middle-aged cohort (45-55 year old)	
	Formation	Decay	Formation	Decay	Formation	Decay
female-female	10.9 ± 1.7	12.1 ± 1.8	7.3 ± 0.4	11.1 ± 0.5	6.6 ± 1.0	5.9 ± 0.9
male-male	13.9 ± 1.8	12.2 ± 1.7	7.7 ± 0.5	10.9 ± 0.6	5.2 ± 1.0	4.7 ± 1.0
female-male	3.1 ± 0.7	6.1 ± 0.9	2.3 ± 0.2	4.8 ± 0.3	4.1 ± 0.6	3.1 ±0.5
male-female	6.0 ± 1.4	7.7 ± 1.5	2.3 ± 0.2	5.0 ± 0.3	3.3 ± 0.5	3.4 ±0.5

TABLE III. **Table corresponding to percentages of ego-alter relationships that have formed or decayed from a close bond among peers in three different age cohorts as shown in graph form in Fig. 2 of main text.** Total number of pairs from each group are given in Supplementary Table 1. The error bars are shown with 95 % confidence level.

Ego - alter relationship	Young adult cohort (17-21 year old)		Adult cohort (25-35 year old)		Middle-aged cohort (45-55 year old)	
	Formation	Decay	Formation	Decay	Formation	Decay
female-female	6.3 ± 1.1	3.8 ±0.8	5.4 ± 0.4	4.1 ± 0.3	5.5 ± 0.5	3.9 ± 0.4
male-male	6.8 ± 1.6	6.6 ± 1.6	7.1 ± 0.7	5.8 ± 0.7	7.6 ± 0.9	6.1 ± 0.8
female-male	7.6 ± 1.7	6.3 ± 1.5	6.0 ± 0.6	5.4 ± 0.6	6.6 ± 0.6	5.1 ± 0.5
male-female	7.5 ± 1.3	5.7 ± 1.2	6.8 ± 0.6	4.7 ± 0.5	7.0 ± 0.8	6.3 ± 0.8

TABLE IV. **Table corresponding to percentages of ego-alter relationships that have formed or decayed from a close bond among non-peers in three different age cohorts as shown in graph form in Fig. 3 of main text.** Total number of pairs from each group are given in a corresponding Supplementary Table 2. The error bars are displayed with 95% confidence level.

IV. TEMPORAL CALLING PATTERNS AMONG YOUNG ADULTS

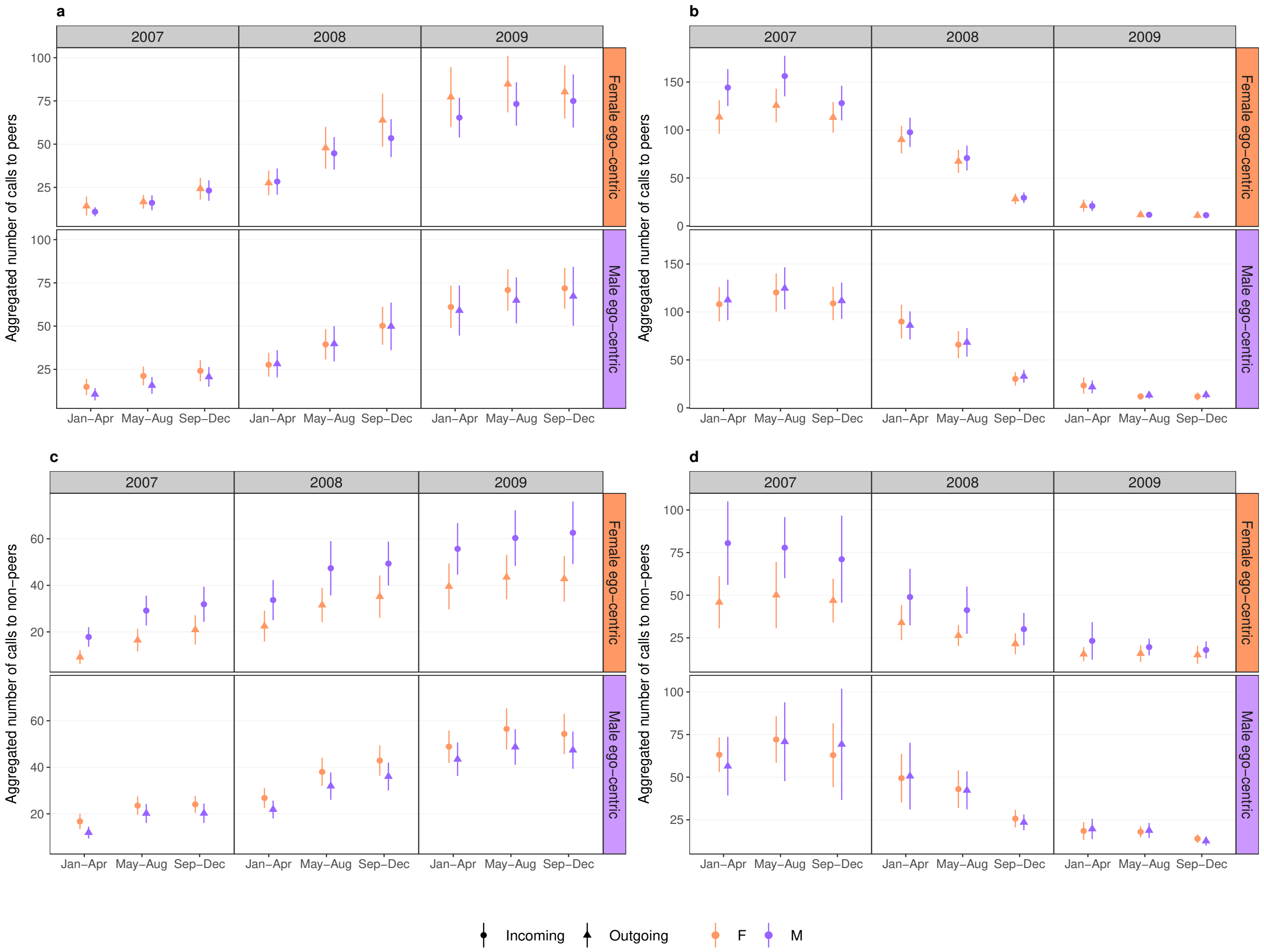

FIG. 2. **Calling activities of young adult age cohorts between peers and non-peers and having interactions with their opposite genders.** The aggregated calling activities of young adult cohorts between their peers (having age differences of less than 10 years) has been shown in (a) and (b) along with non-peers (having age differences of 20 to 40 years) in (c) and (d). The plots on the left exhibit those relationships that form a close bond and the plots on the right exhibit decay of close bonds. The colour and shape schemes are same as the figures in the main text for calling activities of adult and middle-aged cohorts. The calling patterns of the young adult cohorts with both their peers and non-peers have the same characteristics to the adult cohorts except for the decay case in female egocentric links shown in (b) and (d). The males are making distinctively higher number of calls to their female counterparts in decaying relationships.

V. AGE DISTRIBUTION OF THE USERS

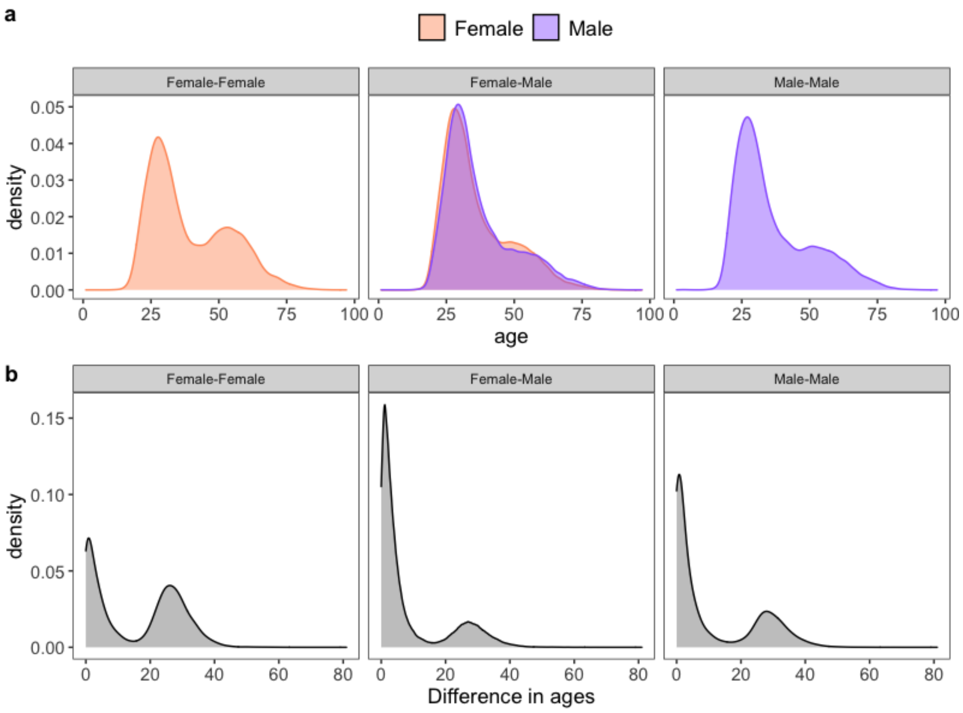

FIG. 3. **Age distribution of the females and males in three different gender based categories of interactions.** (a) The age distribution of males and females in links that exhibit all 178,592 relationships that have been considered in our analysis. The pairs are grouped into three categories consisting of female-female interactions, male-male interactions and female-male interactions. The orange colour represents the age distribution of females while violet represents the distribution for males. (b) The absolute differences of the ages between the callers and the callees is plotted for the three respective categories. In all three of the categories it is seen that the interactions mostly happen between peers (age difference $\leq$ 10 years) and non-peers (age difference between 20 to 40 years of age).

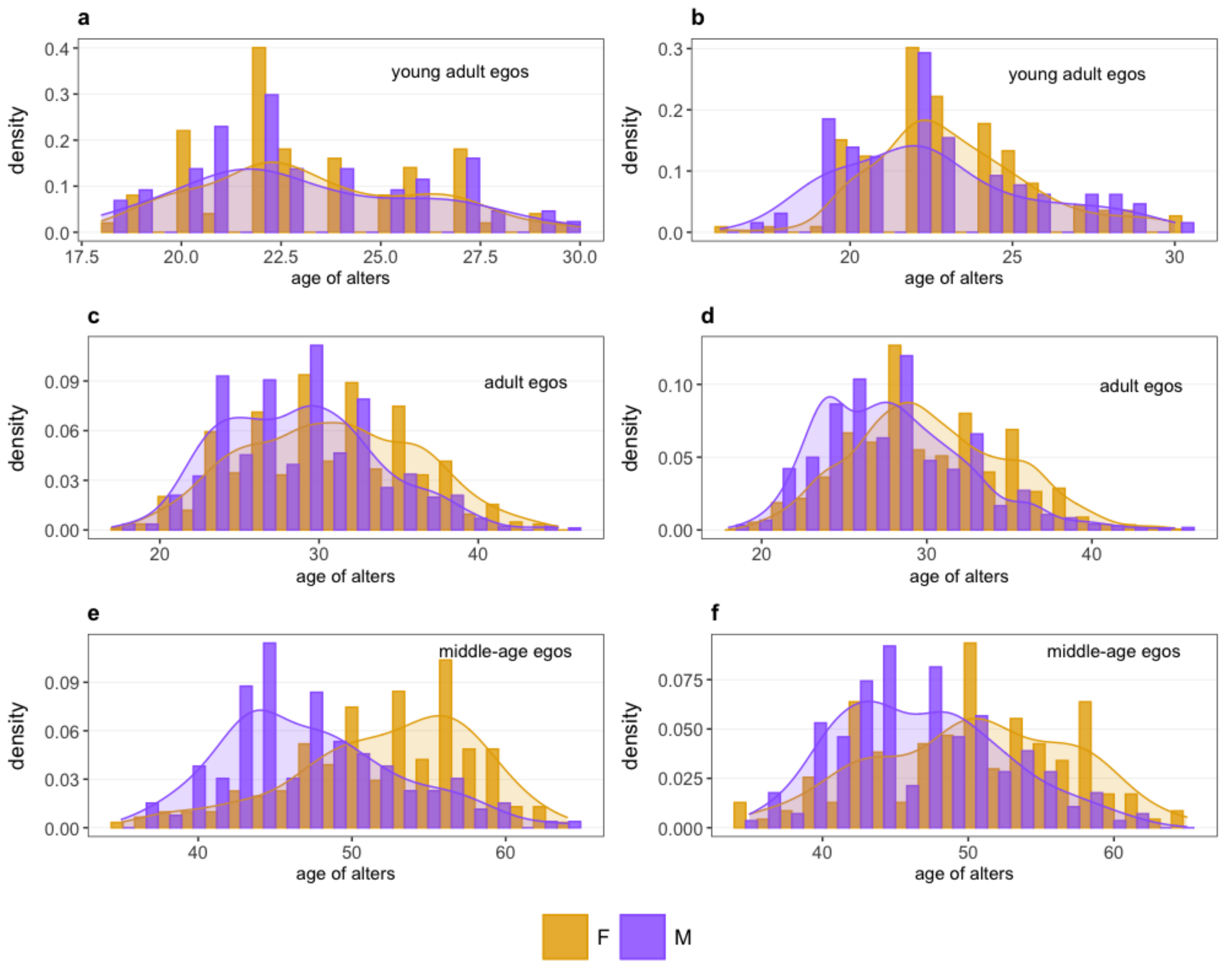

FIG. 4. **Age distribution of the peer alters in the three different age based cohorts for relationships that form and decay.** The age distribution of the alters based on the three different age cohorts of egos (young adult, adult and middle-aged) for relationships that form are shown in (a),(c) and (e) respectively. The distribution for peer alters in decaying relationships for the same cohorts are shown in (b), (d) and (f). The orange colour represents the alters for female egos and violet represents alters for male egos in opposite gender friendships.

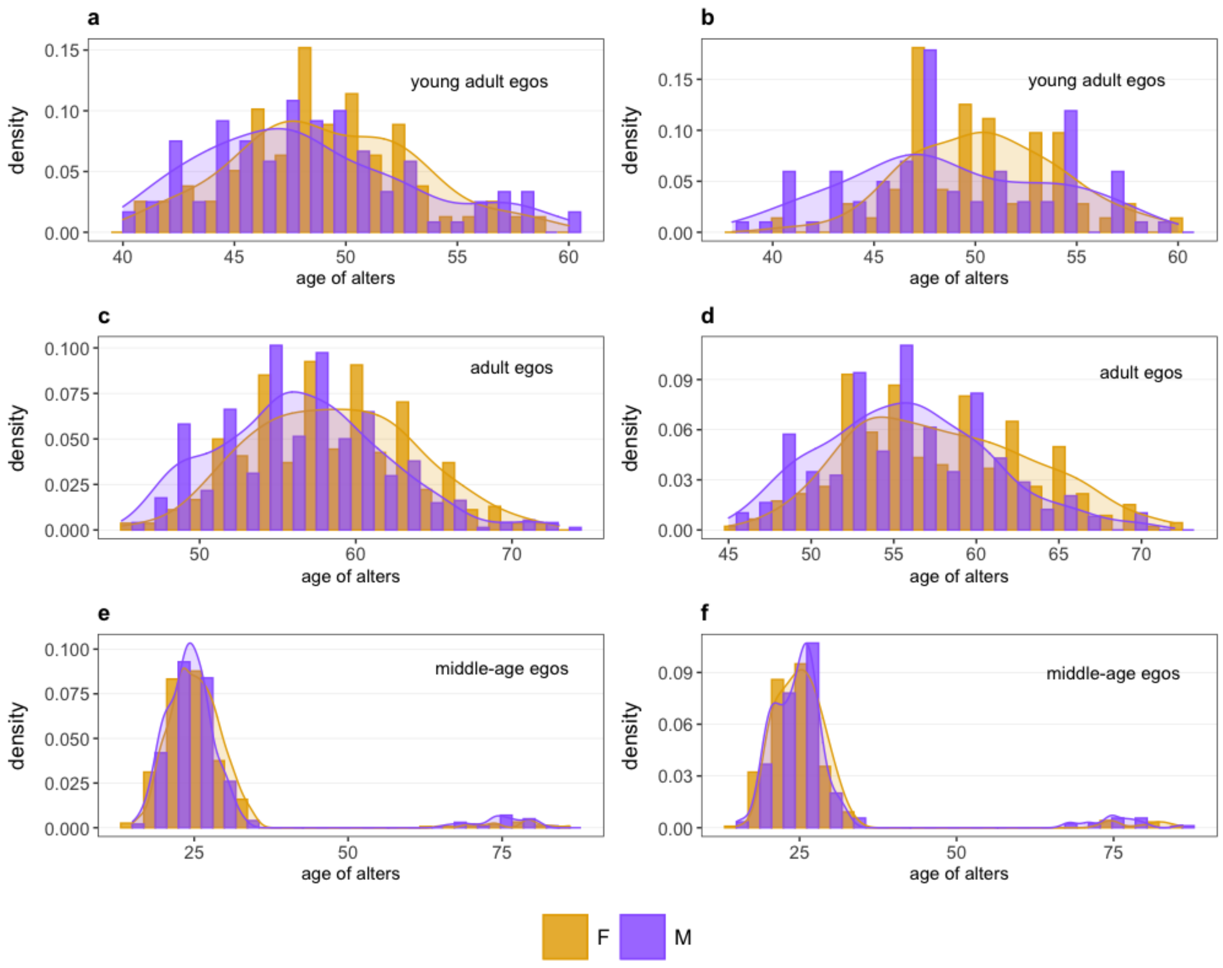

FIG. 5. **Age distribution of the non-peer alters in the three different age based cohorts for relationships that form and decay.** The age distribution of the alters based on the three different age cohorts of egos (young adult, adult and middle-aged) for relationships that form are shown in (a),(c) and (e) respectively. The distribution for non-peer alters in decaying relationships for the same cohorts are shown in (b), (d) and (f). The orange colour represents the alters for female egos and violet represents alters for male egos in opposite gender friendships.
